# Thyroid Function and Thyroid Autoimmunity in Relation to Weight Status and Cardiovascular Risk Factors in Children and Adolescents: A Population-Based Study

**DOI:** 10.4274/jcrpe.2687

**Published:** 2016-06-06

**Authors:** Emilio García-García, María A. Vázquez-López, Eduardo García-Fuentes, Rafael Galera-Martínez, Carolina Gutiérrez-Repiso, Icíar García-Escobar, Antonio Bonillo-Perales

**Affiliations:** 1 Hospital Torrecárdenas, Clinic of Pediatrics, Almeria, Spain; 2 Hospital Carlos Haya, Clinic of Endocrinology and Nutrition, Malaga, Spain

**Keywords:** Thyrotropin, hyperthyrotropinemia, obesity, overweight, child

## Abstract

**Objective:** In obese subjects, slight increases have been observed in thyrotropin [thyroid-stimulating hormone (TSH)] levels, but data in children are scarce. The aim of this study was to evaluate whether thyroid function and autoimmunity vary with weight status in a healthy population of children and adolescents and to determine whether hyperthyrotropinemia is associated with any cardiovascular risk factor.

**Methods:** This cross-sectional epidemiological study was conducted in Almería (Spain) on a representative sample of 1317 healthy subjects aged 2-16 years. Thyroid function, thyroid autoimmunity and cardiovascular risk factors were measured. Chi-square test, analysis of variance and multiple linear regression were used in the statistical analyses.

**Results:** The obese children and adolescents had thyrotropin levels (mean ± standard deviation) of 3.12±2.44 mU/L. These levels were higher than those of overweight subjects (2.79±1.51 mU/L) and of normal weight subjects (2.73±1.30 mU/L) (p=0.02). Levels of free thyroxine and urinary iodine did not differ significantly between the groups. The prevalence (95% confidence interval) of thyroid autoimmunity was lower in the individuals with normal weight (2.9%; 2.0-4.2) than in the overweight (6.3%; 3.9-9.9) and obese subjects (5.6%, 2.5-11.3) (p=0.02). TSH levels were associated with obesity (β=0.36; p<0.001) and thyroid autoimmunity (β=1.10; p<0.001). They were not associated with any cardiovascular risk factor.

**Conclusion:** Obese children and adolescents had higher levels of thyrotropin than those who were overweight and of normal weight. The differences among the groups were of very little clinical significance and could possibly be linked to the higher prevalence of thyroid autoimmunity in obese subjects. The hyperthyrotropinemia in these subjects was not associated with any cardiovascular risk factor.

## WHAT IS ALREADY KNOWN ON THIS TOPIC?

In obese subjects, slight increases have been observed in thyrotropin, or thyroid-stimulating hormone levels.

## WHAT THIS STUDY ADDS?

Populational studies about this topic have been conducted only in adults. To the best of our knowledge, no previous populational study in this field has been conducted in pediatrics.

## INTRODUCTION

The mechanisms that interrelate thyroid function and body weight status are still not completely understood. Hyperthyroid patients lose a lot of weight and regain it when treated. However, in replacement therapy for hypothyroidism, the weight loss is very modest and due more to water deficit than to changes in body fat. Moreover, treatment of obese euthyroid patients with levothyroxine does not induce weight changes (1,2,3,4,5).

In obese subjects, slight increases have been observed in thyrotropin, or thyroid-stimulating hormone (TSH), but these are far from being the cause of overweight and could rather be a consequence of it (1,2,3,4,5). Although the origin of this increase in TSH in obese subjects is not fully understood and may be multifactorial, rather than being an indicator of subclinical hypothyroidism or of thyroid hormone resistance, it is currently considered to be an adaptive change, an attempt to increase basal metabolism in order to avoid further weight gain. This conclusion has been derived from longitudinal studies of children, in which those who lost weight by modifying their lifestyles were observed to decrease their levels of TSH (4,6,7,8,9), while those treated with levothyroxine to normalize TSH did not lose weight (4,8). This adaptive hyperthyrotropinemia is accompanied by a slight increase in free triiodothyronine (T3) and by absence of antithyroid antibodies (4,10,11).

Increases in TSH and T3 in obese subjects could be mediated in part by leptin (12). This hormone, produced by adipose tissue, promotes the synthesis of thyrotropin-releasing hormone in the hypothalamic paraventricular nucleus and the conversion of thyroxine (T4) to T3 in the peripheral tissues (2,4,13), which in turn is the direct result of the action of TSH (14). A low-grade inflammatory reaction mediated by interleukins and other adipokines may also be involved in these changes. In obese subjects, an ultrasound thyroid pattern of chronic low-grade inflammation (irregular hypoechoic areas) unaccompanied by any signs of autoimmunity and termed as “non-autoimmune thyroiditis from obesity” has been reported (1,2,3,5,15).

The aims of this study were to examine whether thyroid function and thyroid autoimmunity vary with weight status in a healthy population of children and adolescents and to determine whether hyperthyrotropinemia in obesity is associated with any cardiovascular risk factor.

## METHODS

This observational cross-sectional epidemiological study was conducted on a population of children aged 2-16 years living in Almeria (southern Spain). A representative sample of 1317 children and adolescents was analyzed. The selection criteria were described in a previous study (16).

The study was carried out in accordance with the guidelines of the 1975 Declaration of Helsinki and was approved by the Research and Ethics Committees of Torrecárdenas Hospital. Written informed consent of the parents or tutors and of the individuals themselves (if they were older than 12) has been obtained after full explanation of the purpose and nature of all procedures used.

Anthropometric measurements were obtained and a physical examination was carried out in all subjects. Wearing light clothing and no shoes, body weight was recorded as the mean of two determinations, using a digital Seca 861 scale with an accuracy of 100 g. Height was recorded as the average of two measurements, measured to the nearest millimeter using a height rod attached to the weighing scale, with the child standing upright. Body mass index (BMI) was calculated. Signs of pubertal onset were determined (testes of at least 4 mL in the males and breast buds in the females), as was the presence of goiter. Waist circumference and systolic and diastolic blood pressure were also measured using calibrated equipment and following standard methods. The examinations were made by six physicians who had previously completed a training and standardisation programme. Obesity, overweight, and normal weight were defined according to thresholds proposed for childhood and adolescence by the International Obesity Task Force (17). The definition for excess weight encompassed the first two of these categories.

Under fasting conditions, blood samples were obtained for determination of glucose, insulin levels, and of the lipid profile [triglycerides, total LDL (low-density lipoprotein), HDL (high-density lipoprotein), and cholesterol], in addition to thyroid hormone and thyroid antibody levels. A urine sample was also collected. Serum concentrations of free T4 (normal range 0.9-1.7 ng/dL), TSH (normal range 0.2-4.2 mU/L), antiperoxidase antibodies (normal less than 34 U/mL), and thyroglobulin antibodies (normal values <115 U/mL) were analyzed by chemiluminescence immunoassay (Roche Diagnostics, Basel, Switzerland). Urinary iodine was determined by the Benotti method (18). A positive result for any antibody was considered to indicate thyroid autoimmunity.

Statistical analysis was performed using SPSS 17.0, and the sample size was determined using Epidat 3.0. The qualitative variables are expressed as percentages with a 95% confidence interval, and the quantitative variables as the mean (standard deviation). Among other statistical tests, chi-square, analysis of variance, and multiple linear regression analyses were performed. In all cases, statistical significance was taken as p<0.05.

## RESULTS

The study population was composed of 1317 children and adolescents with a mean age of 8.8 (4.3) years. 48.8% were female and 38.4% pubescent. The following age groups were established: 402 subjects aged 2-6 years, 504 aged 6-12, and 411 aged 12-16 years. Of the 333 individuals invited to take part in the study, 20.2% refused. The rejection rate was higher among the youngest (26.9%) and the oldest (25.3%) age groups than in the intermediate one (8.4%), but did not differ by gender, ethnicity, or geographic area.

In the study group, 1.2% of the children and adolescents presented with excess weight, 9.8% being obese and 21.4% overweight. The prevalence of thyroid autoimmunity in this population was 3.7% (Table 1).

Comparison of the three subgroups defined in terms of weight status (obesity, overweight, and normal weight) showed that the obese children and adolescents presented TSH levels (3.12±2.44 mU/L) higher than those of the subjects with overweight (2.79±1.51 mU/L) and also higher than those of the subjects with normal weight (2.73±1.30 mU/L) (p=0.02). Free T4 concentrations were not significantly different among the three subgroups (Table 2). There were also no differences between genders, Tanner stage groups, or age groups (data not shown).

The prevalence of thyroid autoimmunity was significantly lower among subjects with normal weight (2.9%) than in the overweight (6.3%) and obese subjects (5.6%) (p=0.02) (Table 2).

TSH levels showed a correlation with obesity after correction for age, Tanner’s stage, gender, ethnic group, iodine intake, and thyroid autoimmunity. However, the magnitude of the association (linear regression coefficient) was quite low after adjusting for presence of thyroid autoimmunity (Table 3).

Among the excess-weight children and adolescents with hyperthyrotropinemia, none of the clinical and laboratory variables studied (including major cardiovascular risk factors) were significantly different from those of the excess-weight subjects with normal TSH concentrations. After correction for the rest of the variables including weight status, thyrotropin was not associated with any of the cardiovascular risk factors (Table 4).

## DISCUSSION

In our study population, the obese children and adolescents had slightly higher levels of thyrotropin, a finding which is in accord with most recent publications (3,6,10). However, population-based studies with relatively large numbers of subjects have been conducted only in adults. Thus, one study carried out in the United States analyzed 3114 subjects, relating BMI and waist circumference with elevated TSH levels (19), while in a Norwegian publication conducted on 1500 individuals, weight gain was associated over time with this elevation (20). However, these findings are not unanimous: a population-based study on adults in our own region and also other studies reported absence of any relationship between thyrotropin and BMI (21,22,23). Increased BMI has also been associated with slightly elevated free T3 (10,19,22,23) and with a slight decrease in free T4 (23,24).

To the best of our knowledge, no previous population-based study in this field has been conducted in pediatric age groups. Reports have been published of samples of obese children and adolescents, most of them from outpatient clinics, presenting a positive association between BMI and TSH (3,6,25,26,27,28,29,30,31,32,33), a positive association with free T3 (3,6,7,14,26,27), and a negative one between free T4 and waist circumference (26) and between free T4 and visceral fat assessed by ultrasound (34). In accordance with our own findings, none of these studies have reported any significant difference by gender or pubertal status (3).

According to these earlier studies, differences in TSH values between obese and normal weight individuals range from 0.2 to 0.8 mU/L, among both adults and children (3,7,10). Given the fact that current clinical practice guidelines recommend treatment when thyrotropin levels exceed 10 mU/L, the reported increased concentrations are not clinically significant (35).

Whether the differences in TSH concentrations are due to a poor iodine status or to a higher frequency of autoimmunity associated with excess weight remains to be determined. No relationship between iodine intake and weight status has been found in this present study nor has it been reported in any previous studies (30). However, the prevalence of thyroid autoimmunity is known to increase in obese children and adolescents (30) and in obese adults (13,21,36). In accordance with other authors, we believe that autoimmunity might be the main factor responsible for the increased concentration of TSH in obese subjects, as the correlation between thyrotropin and BMI is weaker when correcting for it (21). However, other studies on children and adolescents, an age range in which thyroid autoimmunity is much less frequent than in adults, have reported that only a small proportion (<10%) of obese subjects present elevated TSH levels (27,28).

In our opinion, whether or not increased concentrations of thyrotropin in obese subjects without autoimmunity are statistically significant, their clinical significance is negligible. Most previous studies corroborate our findings, in that no cardiovascular risk factor is aggravated, nor is there any increase in the index of insulin resistance in children with hyperthyrotropinaemia (2,7,27,37). This leads us to consider the condition a physiological one and to discard the possibility of using levothyroxine to lower TSH levels in overweight or obese children and adolescents in order to reduce associated comorbidities. On the other hand, there are studies which report a significant association between thyrotropin and metabolic syndrome (38,39), carbohydrate intolerance (24), elevated total and LDL cholesterol (21,40), and elevated triglycerides (24,29,33,40) as well as between low levels of free T4 and increased insulin concentration (34).

In our study population, the obese group of children and adolescents had slightly higher TSH levels than the overweight and normal weight subjects, but these differences, although statistically significant, were of very little or no clinical significance. The increased concentration of TSH is also not associated with any cardiovascular risk factor. This study has also shown that the prevalence of thyroid autoimmunity is higher in children and adolescents with excess weight. The hyperthyrotropinaemia associated with obesity could possibly be linked to a state of autoimmunity.

**Ethics**

Ethics Committee Approval: Research and Ethics Committees of Torrecárdenas Hospital, Almeria, Spain, Informed Consent: It was taken.

Peer-review: External peer-reviewed.

## AUTHORSHIP CONTRIBUTIONS

Concept: Emilio García-García, María A. Vázquez-López, Design: Emilio García-García, María A. Vázquez-López, Data Collection and/or Processing: Eduardo García-Fuentes, Rafael Galera-Martínez, Carolina Gutierrez-Repiso, Icíar García-Escobar, Analysis and/or Interpretation: Emilio García-García, María A. Vázquez-López, Antonio Bonillo-Perales, Literature Research: Emilio García-García, Writing: Emilio García-García.

Financial Disclosure: The authors declared that this study received no financial support.

## Figures and Tables

**Table 1:**
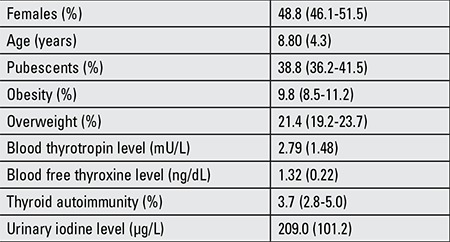
Characteristics of the study sample. The qualitative variables are expressed as percentages (%) with a 95% confidence interval, and the quantitative variables as means ± standard deviation

**Table 2:**
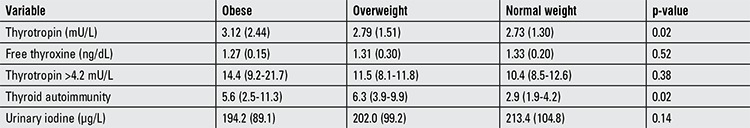
Comparison of variables in three subgroups of children and adolescents by weight status. The qualitative variables are expressed as percentages (%) with a 95% confidence interval, and the quantitative variables as means ± standard deviation

**Table 3:**
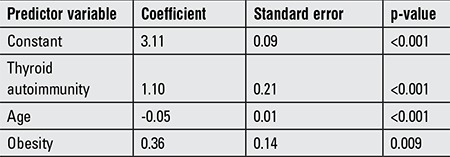
Multiple linear regression model showing the relationship between thyrotropin (response variable) and predictor variables

**Table 4:**
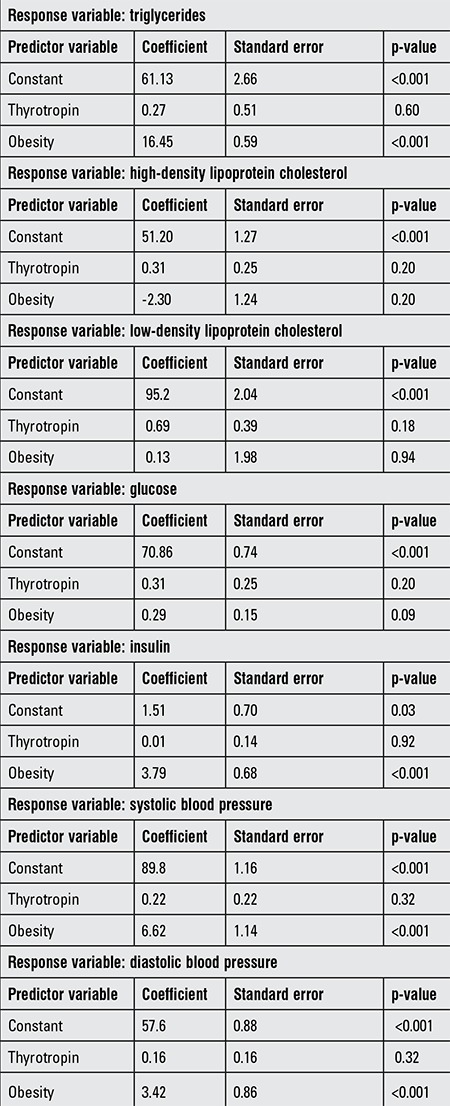
Multiple linear regression models showing the relationship between any cardiovascular risk factor (response variable) and thyrotropin and obesity as predictor variables
